# Validity and inter-rater reliability of medio-lateral knee motion observed during a single-limb mini squat

**DOI:** 10.1186/1471-2474-11-265

**Published:** 2010-11-16

**Authors:** Eva Ageberg, Kim L Bennell, Michael A Hunt, Milena Simic, Ewa M Roos, Mark W Creaby

**Affiliations:** 1 Department of Orthopedics, Clinical Sciences Lund, Lund University, Sweden; 2 Department of Health Sciences, Lund University, Sweden; 3 Centre for Health, Exercise and Sports Medicine, Melbourne School of Health Sciences, University of Melbourne, Melbourne, Victoria, Australia; 4 Department of Physical Therapy, University of British Columbia, Vancouver, British Columbia, Canada; 5 Institute of Sports Science and Clinical Biomechanics, University of Southern Denmark, Odense, Denmark; 6 Centre of Physical Activity Across the Lifespan, School of Exercise Science, Australian Catholic University, Brisbane, Queensland, Australia

## Abstract

**Background:**

Muscle function may influence the risk of knee injury and outcomes following injury. Clinical tests, such as a single-limb mini squat, resemble conditions of daily life and are easy to administer. Fewer squats per 30 seconds indicate poorer function. However, the quality of movement, such as the medio-lateral knee motion may also be important. The aim was to validate an observational clinical test of assessing the medio-lateral knee motion, using a three-dimensional (3-D) motion analysis system. In addition, the inter-rater reliability was evaluated.

**Methods:**

Twenty-five (17 women) non-injured participants (mean age 25.6 years, range 18-37) were included. Visual analysis of the medio-lateral knee motion, scored as knee-over-foot or knee-medial-to-foot by two raters, and 3-D kinematic data were collected simultaneously during a single-limb mini squat. Frontal plane 2-D peak tibial, thigh, and knee varus-valgus angles, and 3-D peak hip internal-external rotation, and knee varus-valgus angles were calculated.

**Results:**

Ten subjects were scored as having a knee-medial-to-foot position and 15 subjects a knee-over-foot position assessed by visual inspection. In 2-D, the peak tibial angle (mean 89.0 (SE 0.7) vs mean 86.3 (SE 0.4) degrees, p = 0.001) and peak thigh angle (mean 77.4 (SE 1.0) vs mean 81.2 (SE 0.5) degrees, p = 0.001) with respect to the horizontal, indicated that the knee was more medially placed than the ankle and thigh, respectively. Thus, the knee was in more valgus (mean 11.6 (SE 1.5) vs 5.0 (SE 0.8) degrees, p < 0.001) in subjects with the knee-medial-to-foot than in those with a knee-over-foot position. In 3-D, the hip was more internally rotated in those with a knee-medial-to-foot than in those with a knee-over-foot position (mean 10.6 (SE 2.1) vs 4.8 (SE 1.8) degrees, p = 0.049), but there was no difference in knee valgus (mean 6.1 (SE 1.8) vs mean 5.0 (SE 1.2) degrees, p = 0.589). The kappa value and percent agreement, respectively, was >0.90 and 96 between raters.

**Conclusions:**

Medio-lateral motion of the knee can reliably be assessed during a single-leg mini-squat. The test is valid in 2-D, while the actual movement, in 3-D, is mainly exhibited as increased internal hip rotation. The single-limb mini squat is feasible and easy to administer in the clinical setting and in research to address lower extremity movement quality.

## Background

Muscle function may influence the risk of knee injury and outcomes following injury [1-6]. Clinical tests of muscle function are meant to resemble conditions of daily life and more strenuous activities [7] and are easy to administer in the clinical setting and in research. High-demand tasks such as hop tests, may not be appropriate, nor replicate daily activities, for less physically active individuals. The single-limb mini squat may be more appropriate as it resembles conditions of daily life, such as stair descent.

A lower number of single-limb mini squats in 30 seconds indicate poorer function [8]. However, the quality of movement during functional tasks may also be important, and may encompass an aspect not reflected by tasks measured in distance, height or frequency [9].

One component of movement quality is postural orientation. This involves the ability to maintain an appropriate relationship between the body segments when performing a dynamic task [10]. At the knee, the medio-lateral position relative to the ankle joint during functional activity involving hip and knee flexion is thought to indicate movement quality. A knee-medial-to-foot position, i.e., when the knee is not aligned over the ankle in the frontal plane, is related to an increased risk of anterior cruciate ligament (ACL) injury [11-15], is more common in individuals with ACL injury or patellofemoral pain syndrome (PFPS) than in non-injured controls [12,16-18], and is related to worse patient-reported function after knee injury [9]. Moreover, preventing a medial position of the knee is suggested to reduce the risk of ACL injuries [18-21] and forms an integral component of ACL rehabilitation through neuromuscular training interventions. Therefore, a knee-medial-to-foot position is deemed inappropriate (less optimal), indicating poor postural orientation. A knee-over-foot position, i.e., when the joints in the lower extremity are well aligned, is considered appropriate (optimal), indicating good postural orientation.

The medio-lateral knee motion can be measured quantitatively with modern motion analysis technology. However, valid and reliable observational clinical tests that can be used in large groups of people are needed. The reliability of visual inspection of the medio-lateral knee motion has been tested in clinical tests such as drop-jump landings [21,22], single-limb squats [23], and lateral step downs [23]. In observational tests, the knee-medial-to-foot position is thought to reflect "knee valgus" or "valgus collapse" [22-24]. The validity of such tests, in terms of the lower limb motion that determines the appearance of a knee with and without a medial position in relation to the foot, has not been established.

The aim of this study was to validate an observational clinical test; the single leg mini-squat, for assessing the position of the knee in relation to the ankle joint. This was done by comparing the two- and three-dimensional biomechanics of the lower limb between people who perform the test with a knee-medial-to foot position and those with a knee-over-foot position. In addition, the inter-rater reliability of the clinical test was assessed.

## Methods

### Subjects

Twenty-five subjects (17 women) aged 18-37 years were recruited from the local community in Melbourne, Australia. Participants were excluded if: (i) they reported any pain, injury or problems within the past month (e.g., fracture, knee surgery/injury, disc hernia), (ii) they had any difficulty moving around on the day of testing or (iii) if they reported any co-morbidities limiting completion of the squatting tests, and (iv) if they had a BMI of greater than 34 kg/m^2^. One subject was excluded from analysis as they were clinically assessed as having a knee-lateral-to-foot position during the single-limb mini squat, which the test was not meant to capture in the present study.

Subject characteristics, including physical activity and self-reported outcomes assessed by the Knee injury and Osteoarthritis Outcome Score (KOOS) [25] are given in Table 1. There were no differences in subject characteristics between participants with a knee-over-foot position and those with a knee-medial to-foot position (Table 1).

**Table 1:** Characteristics of the subjects

Characteristic	Knee-over-foot (n = 15)	Knee-medial-to-foot(n = 10)	All (n = 25)
Age (y), mean (SD)	26 (6.1)	25 (4.1)	26 (5.3)
Women (n)	10	7	17
BMI (kg/m^2^), mean SD	22.5 (3.5)	24.3 (3.9)	23.2 (3.7)
Recreational physical activity/no physical activity (n)	12/3	6/4	18/7
KOOS subscales			
Pain	98 (3.9)	99 (2.6)	98 (3.4)
Symptoms	97 (3.5)	95 (4.1)	96 (3.8)
ADL	100 (0.8)	100 (0.0)	100 (0.6)
Sport/Rec	98 (4.1)	99 (3.4)	98 (3.8)
QOL	97 (7.0)	97 (5.1)	97 (6.2)

BMI, body mass index

The Human Research Ethics Committee at the University of Melbourne approved the study and the participants gave their written informed consent.

### Single-limb mini squat

#### Procedure

Visual analysis of the medio-lateral knee motion and 3-D kinematic data during the single-limb mini squat were collected simultaneously. The right leg was tested in all participants, and the participants were barefoot during the test. Two examiners scored the subjects' knee position in relation to the foot during the observational test. A third examiner, blinded to the clinical scoring of the knee position, collected the 3-D kinematic data. The procedure for the single-limb mini squat test was as follows: A "T" was marked with tape on the floor. The patient stood with the long axis of the foot aligned to the stem of the "T"; the second toe placed on the stem. A bar was placed in front of the participants to provide finger tip support for balance (right and left index fingers). The participant was then asked to look down and bend his/her knee, without bending forward from the hip, until he/she no longer could see the line along the toes (corresponding to about 50 degrees of knee flexion), and then return to extension [26]. The single-limb mini squat was repeated 5 times at a pre-defined speed of 20 squats/min (i.e., 3 seconds from starting position to the knee flexion position and back to the starting position) using a metronome. The other leg was kept with the hip in slight flexion and the knee in about 80 degrees of flexion. Practice trials preceded the measurements.

#### Visual analysis of the medio-lateral knee motion

**Figure 1:**
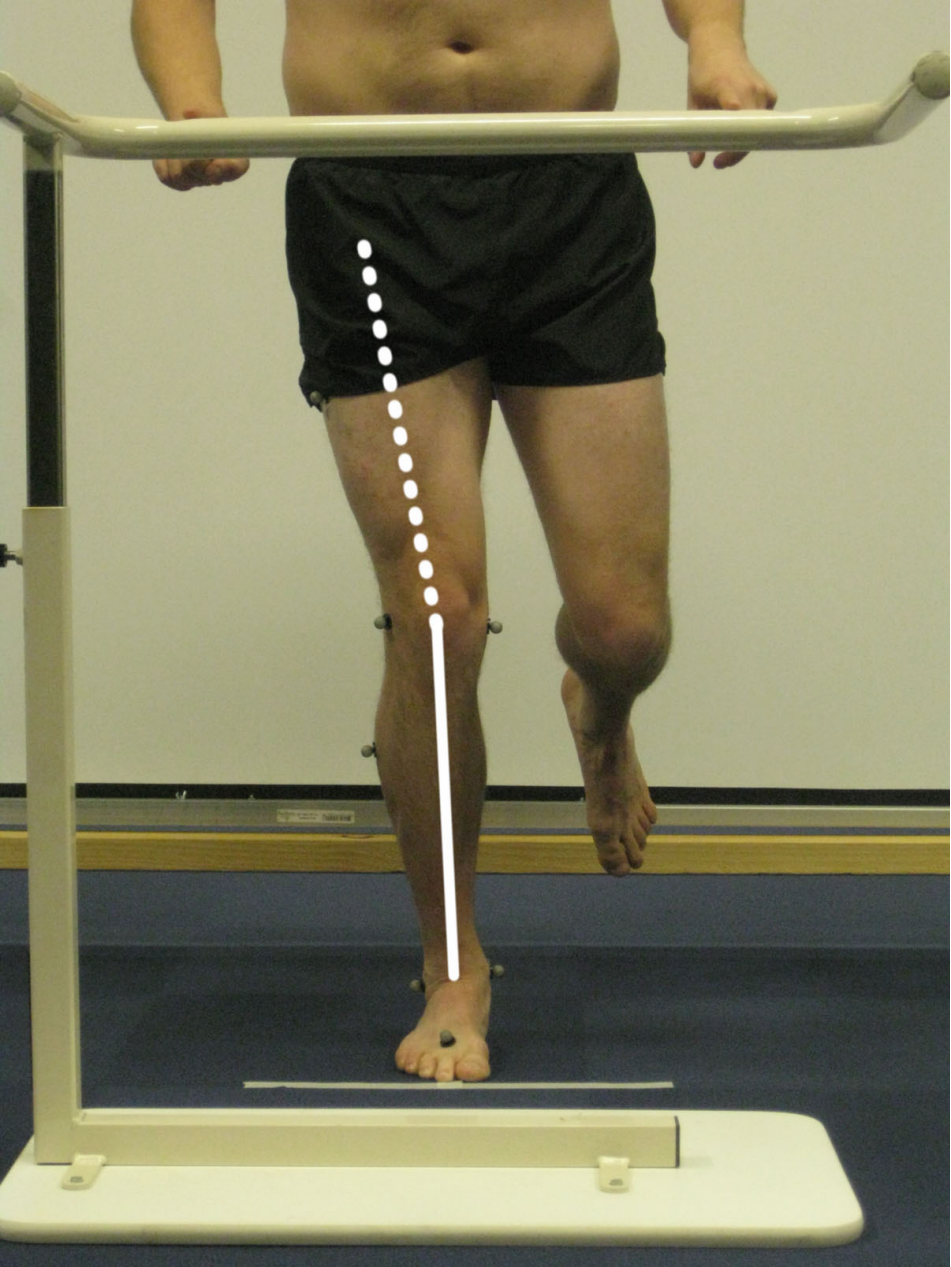
**Knee-over-foot position during the single-limb mini squat**.

**Figure 2:**
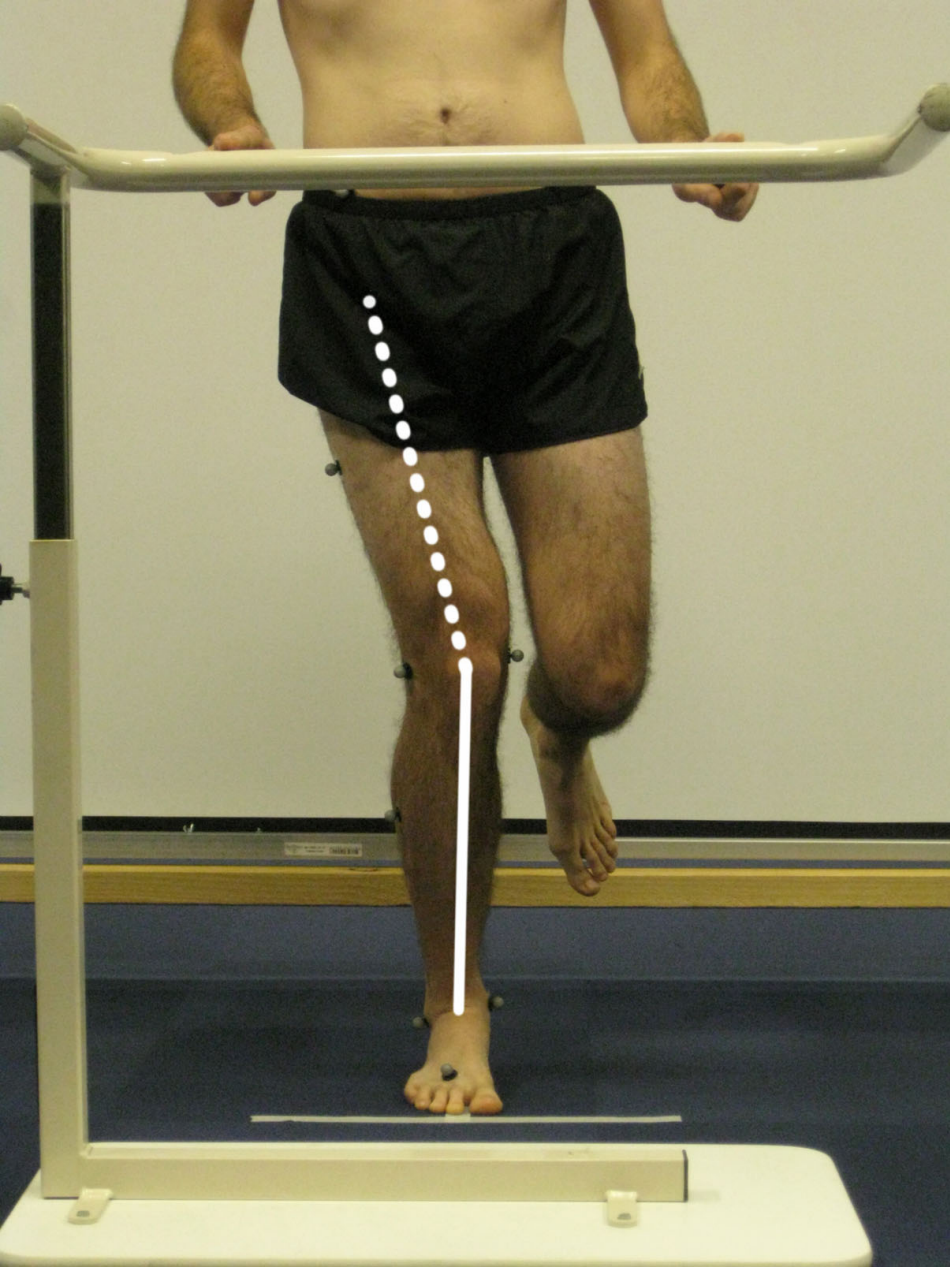
**Knee-medial-to-foot position during the single-limb mini squat**.

During the performance of the single-limb mini squat, the position of the knee in relation to the foot was scored by two musculoskeletal physical therapist researchers (examiners A and B), standing 5 m directly in front of and facing the subject. The examiners had no previous experience of this specific test, but they were well trained by an experienced examiner, from pilot testing preceding the present study. The participants were unaware of what was being assessed during the test. The subject was scored as either having a knee-over-foot position or a knee-medial-to-foot position. A knee-over-foot position was scored when the knee was well aligned over or lateral to the 2^nd ^toe in three or more of five trials (Figure 1, additional file 1). A knee-medial-to-foot position was scored when the knee was placed medial to the 2^nd ^toe in three or more of five trials (Figure 2, additional file 2). This method for rating movement quality was developed by two of the authors (EA and ER; none of them were examiners in the present study); both musculoskeletal physical therapy researchers with more than 15 years of clinical experience within the field.

#### Three-dimensional motion analysis

Three-dimensional kinematic data were collected at 120 Hz using a Vicon motion analysis system with eight M2 CMOS cameras (Vicon, Oxford, UK). The standard Vicon Plug-in-Gait lower-limb marker set was used, and additional markers were attached to the medial knee and ankle during a single static standing trial to determine the relative positioning of joint centers. Reflective markers were attached to the pelvis and right lower limb, for the duration of testing. First, a standing calibration trial was performed.

Two-dimensional angles were computed from the same mini-squat trials as the three-dimensional data, but only the frontal plane coordinates were utilized in the two-dimensional analyses. To compute two-dimensional angles, joint centers for the ankle, knee and hip were defined. The ankle joint centre was defined as the mid-point of the medial and lateral malleolus markers; the knee joint centre was defined as the mid-point of the medial and lateral femoral epicondyle markers. The hip joint centre was defined using the equations of Davis et al [27]. The thigh and shank were defined as straight lines from the hip to knee, and knee to ankle, respectively. The two-dimensional angle of the knee was calculated in the frontal plane of the laboratory coordinate system as the angle between the thigh and shank; a negative angle indicates a valgus position of the knee.

Three-dimensional joint angles (flexion/extension; ab/adduction; internal/external rotation) were computed for the hip and knee using a joint coordinate system approach [28].

Joint angles at the occurrence of peak knee flexion (in 3D) were recorded and the mean of the first 3 mini squats in which the examiners reached consensus were used in statistical analysis.

#### Data analysis

The two examiners observed and scored the subjects simultaneously and separately. After each subject was assessed, the two examiners discussed the scoring of the knee position. If there wasn't agreement between the observers on 3 or more of 5 trials, the single-limb mini squat was repeated until consensus was reached. 2-D peak tibial, peak thigh, and peak knee varus-valgus angles (degrees), and 3-D peak hip internal-external rotation, and peak knee varus-valgus angles (degrees) were calculated and used for validation of the clinical test. The two examiners' scores before consensus were used for inter-rater reliability analysis.

### Statistical analysis

Independent *t*-tests were used to compare 2-D and 3-D data between the subjects with a knee-over-foot position and those with a knee-medial to-foot position. The receiver operating characteristic (ROC) curve (area under the curve) was used to determine the ability of the clinical test to discriminate between those with and without a medial knee position. For inter-rater reliability, the Kappa coefficient, the percent agreement, and the Wilcoxon signed ranks test were used. A kappa value of ≤0.20 was considered poor, 0.21 to 0.40 fair, 0.41 to 0.60 moderate, and >0.60 good agreement [29]. A level of p ≤ 0.05 was chosen to indicate statistical significance.

## Results

### Validity

Ten subjects were scored as having a knee-medial-to-foot position and 15 subjects a knee-over-foot position assessed by visual analysis. There was no difference in peak knee flexion during the squat between the groups (mean 44.6 (SE 2.2) vs 41.9 (SE 1.9) degrees, mean difference -2.7 (95% CI -8.8, 3.3), p = 0.360).

In 2-D, the peak tibial angle (p = 0.001) and peak thigh angle (p = 0.001) were more medially oriented at the knee, and the knee was thus in more valgus (p < 0.001) in subjects with a knee-medial-to-foot position than in those with a knee-over-foot position (Table 2).

**Table 2:** Two- and three-dimensional kinematic data (degrees) for the knee-over-foot and knee-medial-to-foot groups, and between groups.

Kinematic variables (degrees)	Knee-over-foot (n = 15)	Knee-medial-to-foot (n = 10)	Knee-over-foot vs knee-medial to foot	
	
	Mean (SE)	Mean (SE)	Mean difference (95% CI)	p-value
**2-D**				
Peak tibial angle §	86.3 (0.4)	89.0 (0.7)	-2.7 (-4.2, -1.2)	0.001
Peak thigh angle §	81.2 (0.5)	77.4 (1.0)	3.8 (1.7, 5.9)	0.001
Peak knee varus-valgus*	-5.0 (0.8)	-11.6 (1.5)	6.6 (3.4, 9.7)	< 0.001
				
**3-D**				
Peak hip rotation†	4.8 (1.8)	10.6 (2.1)	-5.8 (-11.6, -0.02)	0.049
Peak knee varus-valgus*	-5.0 (1.2)	-6.1 (1.8)	1.1 (-5.5, 3.2)	0.589

* Negative value = valgus, positive value = varus

† Negative value = external rotation, positive value = internal rotation

§ Angles reported relative to the horizontal, with lower values indicating the segment was more medially oriented at the knee

In 3-D, the hip was more internally rotated in those with a knee-medial-to-foot than in those with a knee-over-foot position (p = 0.049). There were no differences between the groups in peak knee varus-valgus angle (Table 2).

**Figure 3:**
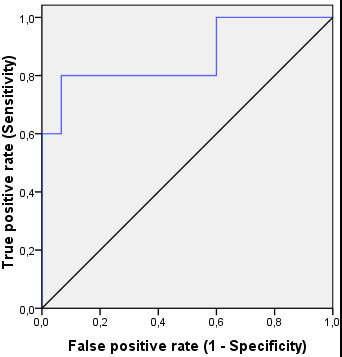
**Receiver Operating Characteristic (ROC) curve linking the examiner ratings with the results from the two-dimensional peak knee varus-valgus angle**. The ROC curve (blue line) moves steeply up and then across, not close to the diagonal (black line), indicating that the observational clinical test is good at discriminating between those with and without a medial knee position.

2-D peak knee varus-valgus angle was used in the ROC analysis, giving an area under the curve of 0.867 (SE 0.082, p = 0.002) (Figure 3).

### Reliability

There was no statistically significant difference between examiners (p = 0.317), indicating no systematic error. The kappa value was 0.92 (95% CI, 0.75 to 1.08), and there was 96% agreement between examiners.

## Discussion

The frontal plane 2-D data indicate that in subjects scored as having a knee-medial-to-foot position during the single-limb mini squat their knee was more medially positioned relative to their hip and ankle, resulting in more 2-D knee valgus than those with a knee-over-foot position. In 3-D, the hip was more internally rotated in subjects with a knee-medial-to-foot position than in those with a knee-over-foot position, but there was no difference between the groups in knee valgus angle. High inter-rater reliability was found for the observational test. These results suggest that the test provides a valid and reliable clinical method to delineate between those with knee-over-foot and knee-medial-to-foot positioning during a single limb mini-squat.

The subjects with a knee-medial-to-foot position displayed a knee valgus angle in 2-D nearly 7 degrees greater than those with a knee-over-foot position. A knee valgus position in 2-D, also called frontal plane knee valgus, has been observed in video analysis studies, assessed by visual inspection [14] or using a digital measuring tool [15]. It is likely that other movements of the lower limb contribute to a frontal plane knee valgus position during movement [24]. This was confirmed in the present study, where the knee valgus position in 2-D was accompanied by a more medially placed tibia and thigh in 2-D, but a greater internal hip rotation in 3-D in those with a knee-medial-to-foot position. It was suggested that the 2-D approach could be used to screen for and evaluate excessive knee valgus [12,30,31]. Because the medio-lateral knee motion assessed by visual inspection during the single-limb mini squat was valid in 2-D, the clinical test may be used as proxy.

The actual movement (in 3-D) for the knee-medial-to-foot position was a greater internal rotation of the hip (about 11 degrees) compared with the knee-over-foot position (about 5 degrees). However, there was no difference between the groups in knee valgus angle in 3-D (mean difference 1.1 degrees). In other words, the appearance of a knee-medial-to-foot position is mainly exhibited as increased internal hip rotation. Thus, a frontal plane knee valgus may not be representative of knee valgus in 3-D.

Greater internal hip rotation has been seen in subjects with patellofemoral pain syndrome compared with controls [32,33]. Our results showed increased internal hip rotation along with greater frontal plane knee valgus. A greater knee valgus movement in 3-D has been reported during functional tests [12,31,34]. In these studies, more strenuous tasks were used [12,31,34], possibly creating a greater demand on the hip stabilizing musculature and, thus, stressing knee valgus movement more than the single-limb mini squat.

A ROC curve was used to assess whether the observational test could discriminate between those with and without a medial knee position. An area under the curve close to 0.5 indicates a poor test, and a value close to 1.0 indicates a good test. The area under the curve for knee valgus in 2-D was reasonably close to 1.0, denoting that the test can discriminate between those with and without a medial knee position.

It has been suggested that the knee-medial-to-foot position is due to poor sensorimotor control. This has been reported, e.g., as a relation between greater internal hip rotation and hip abductor weakness [32,33], and differences in muscle activation patterns of the lower limb and trunk in those with greater compared with smaller knee valgus in 2-D [34]. 2-D valgus anatomical alignment of the knee, measured in standing, was not related to dynamic 2-D knee valgus during a single-limb squat [35], indicating that knee valgus measured statically cannot be used to predict knee valgus during movement. The relative contribution of valgus anatomical alignment, and sensorimotor control that determine a knee-medial-to-foot position during the single-limb mini squat, are subject for further study.

The utility of any assessment tool depends on its validity and reliability. Agreement was good [29], and there was no systematic bias, indicating that visual analysis of the medio-lateral knee motion during single-limb mini squat is reliable between raters. Other studies have failed to report high agreement between observers [23,36]. Possible reasons for this are vague guidelines, and that more than two scoring categories were used [23,36]. The importance of clear and simple standardizations, and adequate rater training, has been highlighted [22,37]. The examiners in the present study received explicit guidelines and thorough training prior to study start, likely contributing to the achieved high reliability. The high reliability also indicates that previous experience of the clinical test is not a necessity for obtaining consistency in measurements.

We have validated a clinical test of assessing the quality of movement by visual analysis. The test resembles conditions of daily life, is easy to administer in the clinical setting and in research, requires no expensive or advanced equipment, and seems to have adequate standardization contributing to high reliability. It also enables the examiner to give immediate feedback to the person being assessed. Further studies may reveal whether the single-limb mini squat can be used as a simple clinical test for screening and evaluation of medio-lateral knee motion in those with or at high risk of knee injury and knee osteoarthritis.

## Conclusions

The medio-lateral knee motion assessed by visual inspection during the single-limb mini squat was valid in 2-D, showing a medially placed tibia and thigh, and knee valgus in individuals with a knee-medial-to-foot position compared to those with a knee-over-foot position. The actual movement, in 3-D, was mainly exhibited as increased internal hip rotation. The inter-rater reliability of the observational clinical test was high. These results suggest that the single limb mini-squat test provides a valid and reliable clinical method to delineate between those with knee-over-foot and knee-medial to-foot positioning. The test is feasible and easy to administer in the clinical setting and in research to address lower extremity movement quality.

## Competing interests

The authors declare that they have no competing interests.

## Authors' contributions

EA and ER contributed to the conception of the study. All authors contributed to the design of the study, participated in data interpretation, and contributed to manuscript revision. MC, MH, and MS collected the data. MC performed data management. EA performed the data analysis, was in charge of data interpretation, and drafted the manuscript. All authors read and approved the final version.

## Pre-publication history

The pre-publication history for this paper can be accessed here:

http://www.biomedcentral.com/1471-2474/11/265/prepub

## Supplementary Material

Additional file 1 **Video showing knee-over-foot position during the single-limb mini squat**. Click here for file

Additional file 2 **Video showing knee-medial-to-foot position during the single-limb mini squat**. Click here for file
